# Infusion with Human Bone Marrow-derived Mesenchymal Stem Cells Improves β-cell Function in Patients and Non-obese Mice with Severe Diabetes

**DOI:** 10.1038/srep37894

**Published:** 2016-12-01

**Authors:** Lirong Li, Hui Hui, Xiaolei Jia, Jie Zhang, Ying Liu, Qianyue Xu, Dalong Zhu

**Affiliations:** 1 Department of Endocrinology, the Affiliated Drum Tower Hospital of Nanjing University Medical School, No. 321, Zhongshan Road, Nanjing, 210008, China; 2 School of Clinical Medicine and Nursing, Suzhou Vocational Health College, Jiangsu, China

## Abstract

Mesenchymal stem cells (MSCs) transplantation is a promising therapeutic strategy for type 1 diabetes (T1D). However, little is known on whether MSC transplantation can benefit T1D patients with ketoacidosis and its potential actions. Here, we show that infusion with bone marrow MSCs preserves β-cell function in some T1D patients with ketoacidosis by decreasing exogenous insulin requirement and increasing plasma C-peptide levels up to 1–2 years. MSC transplantation increased plasma and islet insulin contents in non-obese diabetic (NOD) mice with severe diabetes. In comparison with severe diabetes controls, MSC infusion reduced insulitis, decreased pancreatic TNF-α, and increased IL-10 and TGF-β1 expression in NOD mice. MSC infusion increased the percentages of splenic Tregs and levels of plasma IL-4, IL-10 and TGF-β1, but reduced the percentages of splenic CD8^+^ T and levels of plasma IFN-γ, TNF-α and IL-17A in NOD mice. Finally, infused MSCs predominantly accumulated in pancreatic tissues at 28 days post infusion. The effects of MSCs on preserving β-cell function and modulating inflammation tended to be dose-dependent and multiple doses of MSCs held longer effects in NOD mice. Hence, MSC transplantation preserved β-cell function in T1D patients and NOD mice with severe diabetes by enhancing Treg responses.

Mesenchymal stem cells (MSCs) have capacity of self-renewal and multi-lineage differentiation to form mesodermal, ectodermal and endodermal tissues, including the bone, muscle, neurons, hepatocytes and skin^1^. MSCs can promote angiogenesis and differentiate into insulin producing cells^2^,^3^. Furthermore, MSCs can regulate T cell autoimmunity and inflammation by secreting anti-inflammatory TGF-β1, IL-10, PGE2 and others^4^,^5^. In addition, MSCs can inhibit autoreactive T cell responses, but promote Treg responses^6^. Because of the function and low immunogenicity, allogeneic MSC-based therapies have been tested for their ability to ameliorate autoimmune diseases^7^.

Type 1 diabetes (T1D) results from autoimmune destruction of islet β-cells. Imbalance between pathogenic T cells and regulatory T cells (Tregs) contributes to the pathogenic process of T1D. The continual destruction of islet β-cells leads to very low levels of blood insulin, which fails effectively to maintain euglycemia. Without exogenous insulin, patients with T1D may progress into ketoacidosis, a life-threatening condition. Although exogenous insulin administration can correct hyperglycemia this treatment is insufficient to prevent long-term complications, such as neuropathy, retinopathy and nephropathy. Therefore, preservation of β-cell function in T1D patients, particularly for those with ketoacidosis, is critical for reducing risk to develop chronic diabetic complications.

Previous studies have shown that transplantation with MSCs prevents T1D development in pre-diabetic NOD mice and temporarily reverses hyperglycemia in newly diabetic NOD mice^8^,^9^,^10^. Furthermore, infusion with MSCs preserves β-cell function in human patients with newly diagnosed T1D^11^,^12^,^13^. However, there is no information on whether infusion with bone marrow MSCs can benefit T1D patients with ketoacidosis. Moreover, while infused MSCs can migrate into pancreatic tissues^14^ the dynamic distribution of infused MSCs in a severe diabetic condition is not fully understood. In addition, therapeutic effects of MSC transplantation are associated with modulation of autoimmunity^4^,^5^,^6^, however, the mechanisms underlying the action of infused MSCs in a severe diabetic condition have not been clarified. Moreover, whether the therapeutic effects of MSC transplantation is dose-dependent and whether repeated infusion is necessary for preserving β-cell function are still in debate^15^,^16^.

In this study, we first tested the effects of MSC infusion on β-cell function in T1D patients with ketoacidosis and examined the impact of different doses and frequencies of MSCs on β-cell function and Treg responses in NOD mice with severe T1D. Finally, we characterized the distribution of infused MSCs in NOD mice with severe diabetes longitudinally. Our data indicated that infusion with MSCs preserved β-cell function in some T1D patients with ketoacidosis. Infusion with MSCs improved glucose metabolisms and enhanced Treg responses in NOD mice with severe diabetes. In addition, we provided the evidence that the infused MSCs effectively accumulated in the pancreatic tissues of severe diabetic NOD mice. The therapeutic effects of MSC infusion tended to dose-dependent and repeated infusion with MSCs held longer effects in NOD mice.

## Results

### Infusion with MSCs Preserves β-cell Function in T1D Patients with Ketoacidosis

**Figure 1:**
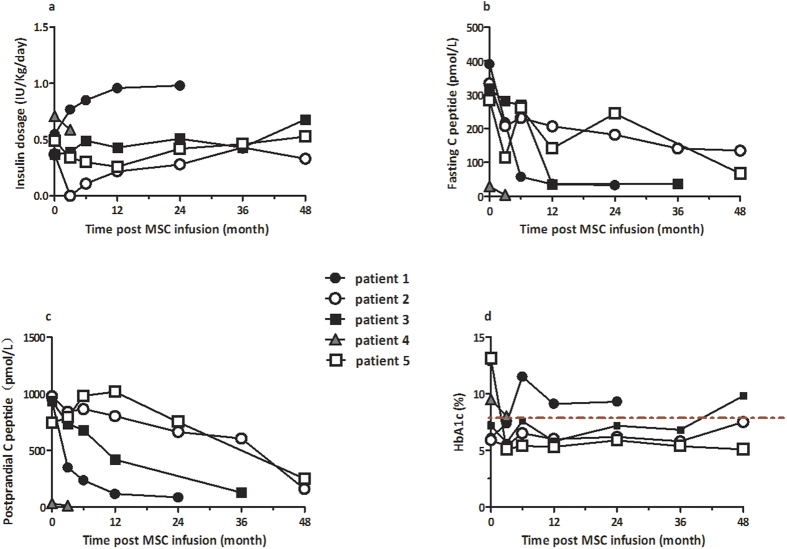
Infusion with MSCs preserves β-cell function in T1D patients with ketoacidosis. A total of five T1D patients with ketoacidosis were recruited and treated with bone marrow MSCs. Mean daily exogenous insulin dosages of requirement for individual patients to control hyperglycemia were recorded and the levels of fasting and postprandial plasma insulin C-peptide as well as HbA1c over the follow-up periods were determined longitudinally. Data are expressed as individual values of the five patients throughout the observation periods. (**a**) The mean daily dosages of exogenous insulin. (**b**) The levels of plasma insulin C-peptide. (**c**) The levels of plasma postprandial C-peptide. (**d**) The levels of blood HbA1c, the horizontal dash line indicate the value of 7.5% for HbA1c. The open symbols in (**a–d**) represent responders while the closed symbols represent non-responders or patients lost.

To determine the potential effect of MSC infusion on T1D patients with ketoacidosis, five T1D patients with ketoacidosis were recruited and their demographics and characteristics are shown in Table 1. Following management for ketoacidosis and infusion with MSCs, those patients were followed up for 4 years. During the observation period, one case was lost to follow up due to personal reasons and there was not a single patient, who developed MSC-related malignancy and side effects. Two out of four patients responded to MSC transplantation by reducing exogenous insulin requirement to control hyperglycemia for 1–2 years and one patient became insulin-independent for three months (Fig. 1a). Although another patient did not reduce exogenous insulin requirement she did not require for an increased dose of insulin for 3 years. Those responders displayed a slow decrease in the levels of plasma C-peptide and one responder increased the levels of postprandial C-peptide for one year (Fig. 1b and c). All responders maintained similar or lower levels of HbA1c for at least three years, as compared with that before treatment (Fig. 1d), an indication of effective control of hyperglycemia. Together, infusion with MSCs preserved β-cell function in some T1D patients with ketoacidosis.

### Infusion with MSCs Preserves β-cell Function in Severe Diabetic NOD Mice

**Figure 2:**
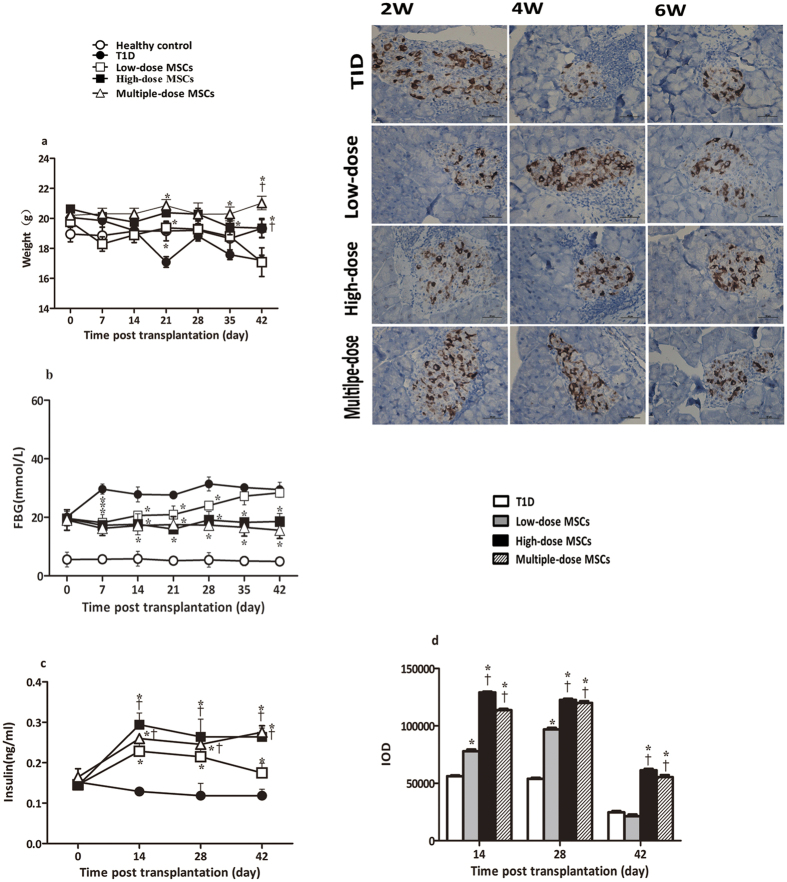
Infusion with MSCs preserves β-cell function in NOD mice with severe diabetes. Following accelerating diabetes development, the mice were randomized and treated with vehicle or low, high or multiple-dose of MSCs. The body weights and the levels of fasting blood glucose and plasma insulin in individual diabetic mice and healthy control mice were measured at the indicated time points post infusion. In addition, some mice from each group of diabetic mice were sacrificed and the contents of islet insulin were determined by immunohistochemistry. (**a**) The body weights. (**b**) The levels of fasting blood glucose. (**c**) The levels of plasma insulin. (**d**) Immunohistochemistry analysis of insulin contents. Data are representative images (magnification x400) or expressed as the mean ± SEM of each group (n = 6 per group per time point for all) of mice at the indicated time points. Statistical significance was analyzed by ANOVA and post hoc Fisher’s least significant difference test in (**a–d**). *P < 0.05 *vs*. the diabetic control. ^†^*P* < 0.05 *vs*. the low-dose group.

To test the effects of MSC transplantation on severe T1D, pre-diabetic female NOD mice were injected with multiple low doses of streptozotocin (STZ) to accelerate T1D development. After the mice developed severe diabetes, they were randomized and infused with vehicle alone as the diabetic controls, with a single low dose, high dose, or multiple doses of MSCs as different treatment groups. The mice, together with a group of C57BL/6 healthy controls, were monitored for their body weights, fasting blood glucose (FBG) and fasting plasma insulin (FPI) longitudinally. As shown in Fig. 2a, while healthy control mice increased body weight slightly throughout the observation period the mice in the diabetic group gradually decreased body weights. Interestingly, the body weights in the mice that had been treated with MSCs were significantly heavier than that in the diabetic controls (*P* < 0.05 for all), and the body weights in the multiple-dose and high dose MSC groups of mice were significantly heavier than that in the low-dose group (*P* = 0.018, *P* = 0.032, respectively) at different time points post treatment. Hence, infusion with MSCs mitigated or prevented long-term hyperglycemia-mediated loss of body weights in NOD mice with severe T1D.

Longitudinal analysis of FBG indicated that the levels of FBG in the low-dose MSC group of mice were significantly lower than that in the diabetic control at 1–4 weeks post treatment (*P* = 0.039, 0.042, 0.046, 0.048, Fig. 2b). The levels of FBG in both the high-dose and multiple-dose MSC groups were significantly lower than that in the diabetic controls throughout the observation period (*P* < 0.05 for all). Further analysis showed that the levels of FPI gradually decreased in the diabetic controls, but significantly increased in all of MSC-infused mice at different time points, as compared with that before treatment (*P* < 0.05 for all). Moreover, the levels of FPI in different groups of MSC-treated mice were significantly higher than that in the diabetic controls throughout the follow-up period (*P* = 0.033, 0.040, 0.047; *P* = 0.018, 0.021 0.030; *P* = 0.023, 0.032, 0.020, respectively). The levels of FPI in the high-dose and multiple-dose MSC groups of mice were significantly higher than that in the low-dose group of mice (*P* = 0.038, 0.043, 0.026*; P* = 0.045, 0.047, 0.029, Fig. 2c). Immunohistochemistry analysis revealed that the intensity of anti-insulin staining in the pancreatic islets from the diabetic controls was gradually reduced and was sustainably lower than that in the mice that had been treated with MSCs (Fig. 2d). The intensity of anti-insulin staining in the islets from the high-dose and multiple-dose MSC groups was significantly higher than that in the low-dose MSCs group (*P* = 0.033, 0.043, 0.037; *P* = 0.038, 0.044, 0.040, respectively). Collectively, these four lines of data clearly demonstrated that treatment with MSCs preserved β-cell function and the therapeutic effect trended to be dose-dependent in NOD mice with severe T1D.

### Infusion with MSCs Reduces Insulitis and Modulates Pancreatic Cytokine Expression in NOD Mice with Severe T1D

**Figure 3:**
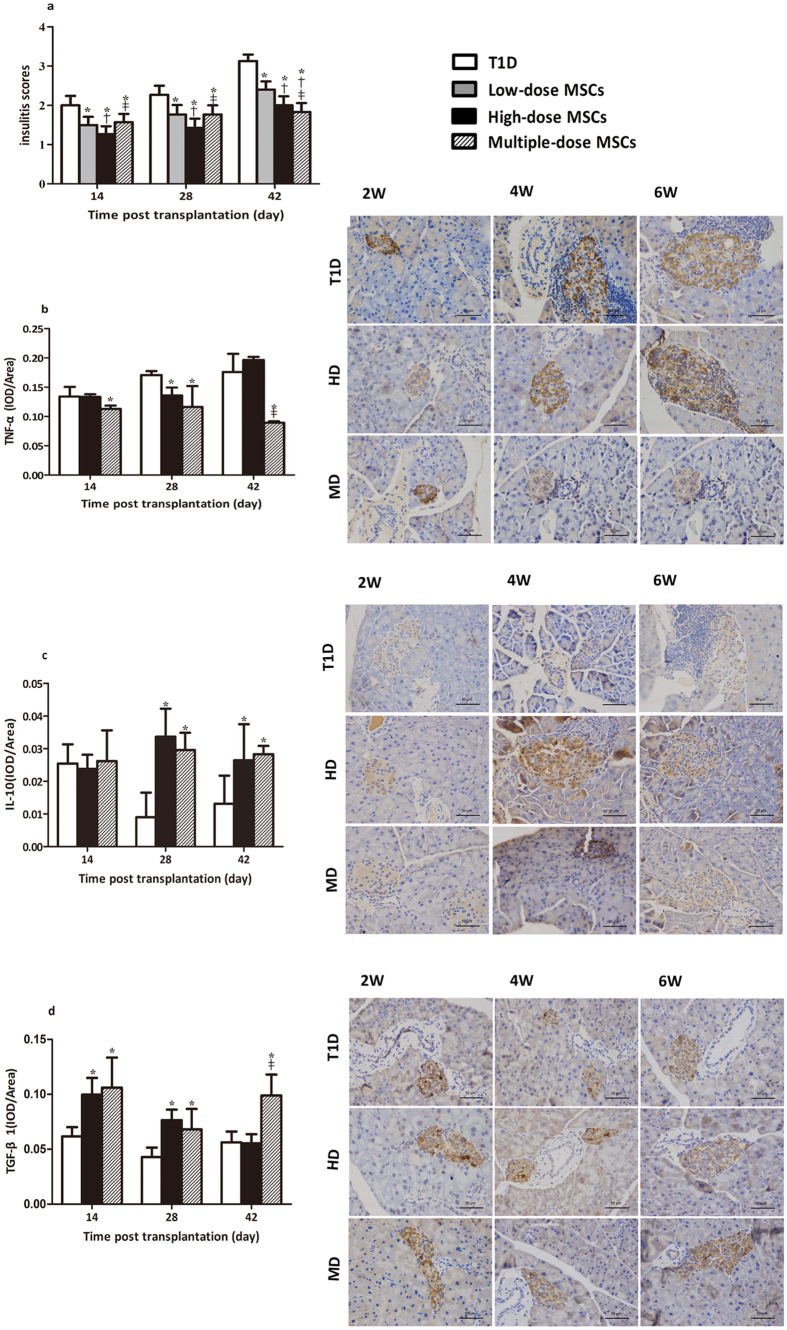
Infusion with MSCs reduces insulitis and modulates cytokine levels in the pancreatic islets of NOD mice. Some mice from each group were sacrificed at the indicated time points post MSC infusion. The degrees of insulitis and the levels of TNF-α, IL-10 and TGF-β1 expression in the pancreatic islets were determined by histology and immunohistochemistry and quantitatively analyzed. (**a**) The insulitis scores. (**b**), (**c**) and (**d**) Immunohistochemistry and quantitative analysis of TNF-α, IL-10, and TGF-β1 expression in the pancreatic islets, respectively. Data are representative images (magnification x200) or expressed as the mean ± SEM of individual groups of mice (n = 6 per group per time points). Statistical significance was analyzed by ANOVA and post hoc Fisher’s least significant difference test in (**a–d**). *P < 0.05 *vs*. the diabetic control. ^†^*P* < 0.05 *vs*. the low-dose group. ^‡^*P* < 0.05 *vs*. the high-dose group.

MSCs can secrete cytokines to inhibit inflammation. To understand the therapeutic effect of MSCs, the degrees of insulitis in the different groups of mice were determined by histology. The insulitis scores in the mice that had been treated with MSCs were significantly lower than that in the diabetic controls (*P* < 0.05 for all) and the insulitis scores in the high-dose MSC group were significantly lower than that in the low–dose MSC group at all time points tested (*P* = 0.041, 0.043, 0.039, Fig. 3a). Furthermore, the insulitis scores in the high-dose MSC group were significantly lower than that in the multiple-dose MSC group at 14 and 28 days post treatment (*P* = 0.040, *P* = 0.044, respectively), but significantly higher than that in the multiple-dose MSC group at 42 days post treatment (*P* = 0.046). These data indicated that the inhibitory effects of MSC infusion trended to be dose-dependent and last longer in the mice that had been treated with multiple-dose of MSCs. Immunohistochemistry analysis of the levels of pancreatic cytokines indicated that the levels of TNF-α in the high-dose and multiple-dose MSC groups were significantly lower than that in the diabetic controls (*P* = 0.045, 0.048, 0.039, 0.026, respectively, Fig. 3b), but the levels of IL-10 and TGF-β1 were significantly higher than that in the diabetic controls at different time points post treatment tested (*P* = 0.011, 0.034, 0.016, 0.031*, P* = 0.045, 0.047, 0.041, 0.048, 0.039, respectively, Fig. 3c and d). Furthermore, the levels of TNF-α in the multiple-dose MSC group were significantly lower than that in the high-dose MSC group, but the levels of TGF-β1 were significantly higher than that in the high-dose MSC group at 42 days post treatment (*P* = 0.028, 0.031, respectively). Further analysis revealed that the levels of pancreatic IL-10 were positively correlated with the levels of FPI in all of the MSC-treated mice at 28 and 42 days post treatment (*r* = 0.991, *P* = 0.045). These data further supported that the therapeutic effect of MSC infusion were dose-dependent and that of multiple-dose MSC transplantation lasted longer in severe T1D mice.

### Infusion with MSCs Increases the Frequency of Splenic Tregs in NOD Mice with Severe T1D

**Figure 4:**
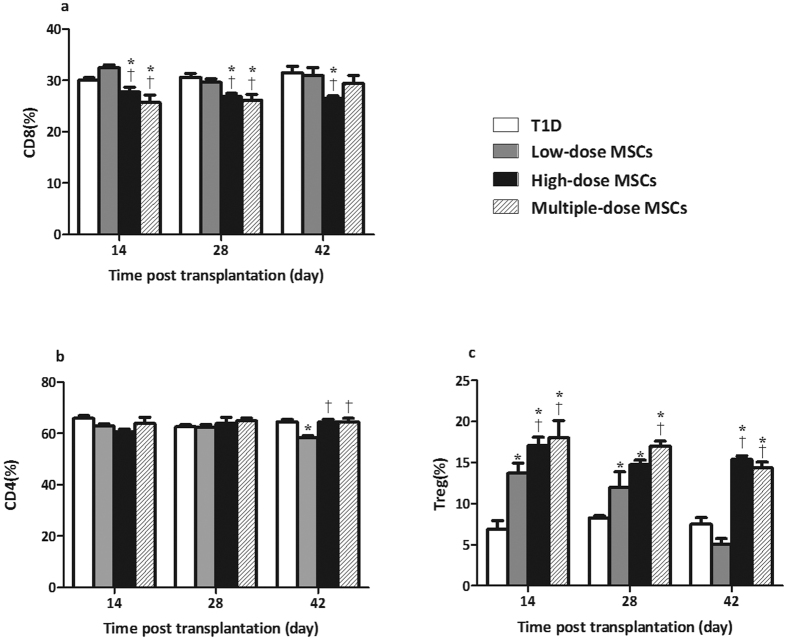
Infusion with MSCs increases the frequency of splenic Tregs in diabetic NOD mice. The percentages of splenic CD4^+^, CD8^+^ T cells and CD4^+^CD25^+^Foxp_3_^+^ Tregs in individual mice were determined by flow cytometry. (**a**) The frequency of splenic CD8^+^ T cells; (**b**) The frequency of splenic CD4^+^ T cells. (**c**) The frequency of splenic Tregs in total CD4^+^ T cells. Data are expressed as the mean ± SEM of individual groups of mice (n = 6 per group per time points). Statistical significance was analyzed by ANOVA and post hoc Fisher’s least significant difference test in (**a**–**c**). *P < 0.05 vs. the diabetic control. ^†^*P* < 0.05 *vs*. the low-dose group.

MSCs can promote Treg responses, which potently inhibit T cell autoimmunity. To further understand the mechanisms underlying the action of MSCs, the frequency of splenic CD4^+^, CD8^+^ T cells, and CD4^+^CD25^+^Foxp_3_^+^ Tregs in the different groups of mice was characterized longitudinally by flow cytometry. The percentages of CD8^+^ T cells in the high-dose and multiple-dose MSC groups were significantly lower than that in the diabetic controls and low-dose MSC groups at the indicated time points tested (*P* < 0.05 for all, Fig. 4a). Furthermore, the percentages of splenic CD4^+^ T cells in the high-dose and multiple-dose MSC groups were significantly higher than that in the low-dose MSC group at 42 days post treatment (*P* = 0.043, *P* = 0.046, respectively, Fig. 4b). In addition, the percentages of splenic Tregs in most groups of mice that had been treated with MSCs, except for the low-dose MSC group at 42 days post treatment, were significantly higher than that in the diabetic controls (*P* = 0.030, 0.022, 0.016, 0.033, 0.028, 0,021, 0.012, 0.018, respectively, Fig. 4c). The percentages of splenic Tregs in the high-dose and multiple-dose MSC groups were significantly higher than that in the low-dose MSC group (*P* = 0.040, 0.023; *P* = 0.037, 0.034, 0.025, respectively) at different time points post treatment. Clearly, infusion with MSCs enhanced Treg responses in NOD mice with severe T1D in a dose- or frequency-dependent manner.

### Infusion with MSCs Modulates Plasma Cytokine Levels in NOD Mice with Severe T1D

**Figure 5:**
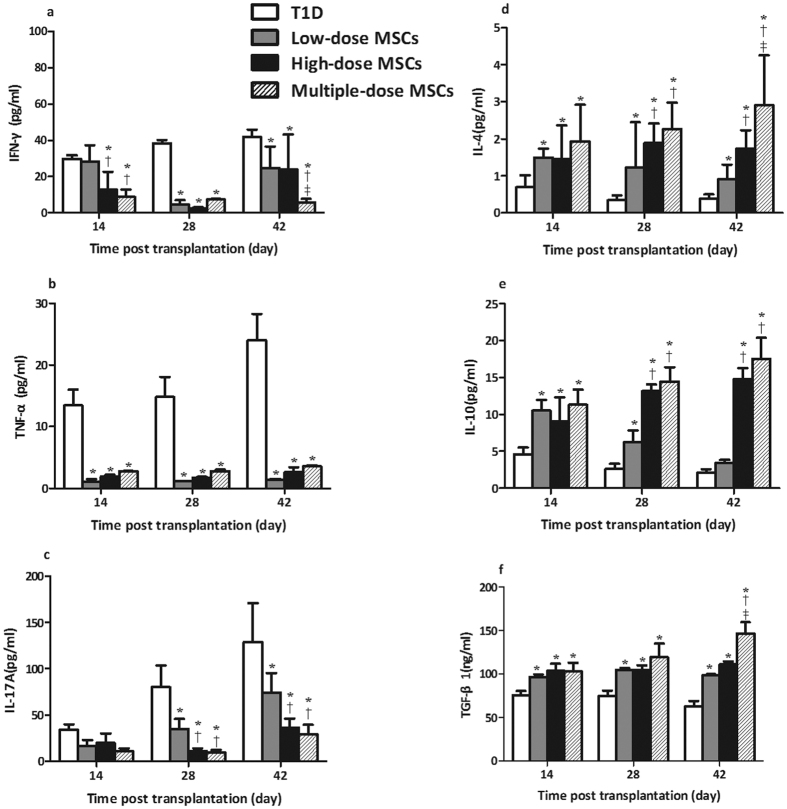
Infusion with MSCs modulates plasma cytokine levels in diabetic NOD mice. The levels of plasma IFN-γ, TNF-α, IL-17A, IL-4, IL-10 and TGF-β1 in individual mice at the indicated time points post infusion were determined. (**a**) The levels of plasma IFN-γ. (**b**) The levels of plasma TNF-α. (**c**) The levels of plasma IL-17A. (**d**) The levels of plasma IL-4. (**e**) The levels of plasma IL-10. (**f**) The levels of plasma TGF-β1. Data are expressed as the mean ± SEM of individual groups of mice (n = 6 per group per time points). Statistical significance was analyzed by ANOVA and post hoc Fisher’s least significant difference test in (**a–f**). *P < 0.05 *vs*. the diabetic control. ^†^*P* < 0.05 *vs*. the low-dose group. ^‡^*P* < 0.05 *vs*. the high-dose group.

Systemic cytokine levels affect the pathogenic process of T1D. Next, the levels of plasma IFN-γ, TNF-α, IL-17A, IL-4, IL-10 and TGF-β1 in the different groups of mice were examined longitudinally by immunology multiplex assay. The levels of plasma pro-inflammatory IFN-γ, TNF-α, and IL-17A gradually increased while the levels of plasma anti-inflammatory IL-4, IL-10 and TGF-β1 decreased with time in the diabetic controls (Fig. 5). In comparison with that in the diabetic controls, significantly decreased levels of plasma IFN-γ, TNF-α, and IL-17A, but elevated levels of plasma IL-4, IL-10 and TGF-β1 were detected in the mice that had been treated with MSCs (*P* < 0.05 for all). Furthermore, the levels of plasma IFN-γ and IL-17A in the high-dose and multiple-dose MSC groups were significantly lower than that in the low-dose MSC group (*P* = 0.042, 0.039, 0.031, *P* = 0.040, 0.042, 0.037, 0.035, respectively) while the levels of plasma IL-4, IL-10 and TGF-β1 in the high-dose and multiple-dose MSC groups were significantly higher than that in the low-dose MSC group at some time points post treatment (*P* < 0.05 for all). Notably, the significantly altered plasma cytokine levels were observed in the multiple-dose MSC group, as compared with high-dose group (*P* = 0.032, 0.029, 0.034, respectively). Thus, infusion with MSCs, particularly with multiple-dose MSCs significantly reduced pro-inflammatory cytokine responses, but enhanced anti-inflammatory cytokine responses in NOD mice with severe T1D.

### Infused MSCs Temporarily Accumulates in the Pancreatic Tissues in NOD Mice with Severe T1D

**Figure 6:**
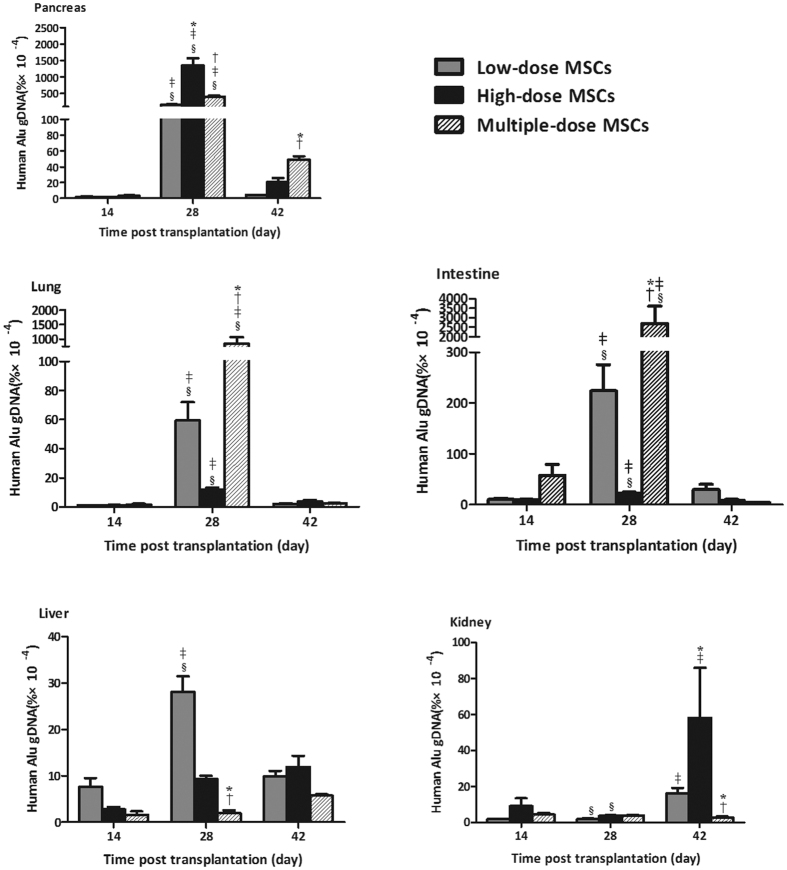
Infused MSCs migrate and accumulate in the pancreatic tissues in diabetic NOD mice. Following infusion with MSCs, some mice from each group were sacrificed and their lung, liver, intestine, kidney and pancreatic tissues were dissected. The levels of human Alu element DNA were determined by quantitative RT-PCR and analyzed, according to the standard curve. Data are expressed as the mean ± SEM of individual groups of mice (n = 6 per group per time points). Statistical significance was analyzed by ANOVA and post hoc Fisher’s least significant difference test. *P < 0.05 *vs.* the low-dose group. ^†^*P* < 0.05 *vs.* the high-dose group. ^‡^*P* < 0.05 *vs*. the mice at 14 days post treatment. ^§^*P* < 0.05 *vs.* the mice at 42 days post treatment.

Finally, the distribution of infused MSCs in the different groups of mice were analyzed by measuring the levels of human Alu DNA using quantitative RT-PCR. The levels of human Alu DNA were detected in the lung, liver, kidney, intestine and pancreatic tissues at two weeks post treatment and increased at four weeks post infusion, followed by declining, except for in the kidney. The levels of human Alu DNA in the intestines were the highest among all the organs tested, and the levels of human Alu DNA in the pancreatic tissues were significantly higher than that in the lung, liver and kidney at four weeks post treatment (*P* < 0.05 for all, Fig. 6). Interestingly, although the levels of human Alu DNA in the pancreatic tissues from the high-dose MSC-infused mice were significantly higher than that in both the low-dose and multiple-dose groups at 28 days post infusion, the levels of human Alu DNA in the multiple-dose group were significantly higher than that in both the low-dose and high-dose groups at 42 days post treatment (*P* = 0.037, 0.040, *P* = 0.013, 0.043, respectively). Further analysis revealed that the levels of human Alu DNA in the pancreatic tissues were positively correlated with the levels of pancreatic IL-10 and TGF-β1 expression in all mice at 42 days post treatment (*r* = 0.723, *P* = 0.028 and *r* = 0.998, *P* = 0.045, respectively). Therefore, the infused MSCs effectively accumulated in the pancreatic tissues, besides the intestine and infusion with multiple doses of MSCs prolonged the presence of MSCs in the pancreatic tissues in NOD mice with severe T1D.

## Discussion

In this study, we tested the effects of bone marrow MSC infusion on five T1D patients with ketoacidosis. Expect for one patients without complete record, two out of four patients responded to the therapies by preserving β-cell function. Evidentially, these two patients required 50% reduced dosages of insulin within one or two years and one patient achieved insulin-independent for three months. Furthermore, these two patients either displayed slowly reduced levels of fasting and post-prandial plasma C-peptide or showed slightly increased levels of post-prandial plasma C-peptide. In contrast, other two non-responding patients exhibited slowly increased dosages of daily insulin and rapidly reduced levels of fasting and post-prandial C-peptide during the follow-up period. To the best of our knowledge, this was the first study of the effect of MSC infusion on patients with severe diabetes and our novel data extended previous findings^11^,^12^,^13^ and support the notion that infusion with MSCs benefits T1D patients by preserving β-cell function. Notably, preservation of β-cell function and control of hyperglycemia are crucial for reducing and preventing long-term hyperglycemia-related complication, which is a leading cause of morbidity and mortality of diabetic patients^17^,^18^. Our findings may aid in design of new therapies for patients with severe T1D.

While infusion with a single-dose of MSCs induces transiently decreases in blood glucose in diabetic mice^19^,^20^ infusion with multiple-dose of MSCs can effectively restore glucose homeostasis by inhibiting oxidative stress and promoting functional β-cell repair in STZ-induced Balb/c and NOD/scid mice^15^,^16^,^21^,^22^. To optimize the therapeutic effect of MSC infusion on severe diabetes, we compared the effects of infusion with different doses of MSCs in NOD mice with severe T1D. We found that infusion with MSCs significantly preserved β-cell function by decreasing the levels of FBG, but increasing the levels of plasma insulin and the contents of islet insulin throughout the 6-week observation period in NOD mice with severe T1D. The therapeutic effects tended to be dose-dependent and infusion with multiple-dose of MSCs displayed longer effect in the mice. These findings are valuable for design of MSC-based immunotherapies for severe diabetes. However, the therapy did not reverse hyperglycemia in this model. The failure may stem from severe diabetes in our experimental model with strong autoimmunity.

It is well known that MSCs can down-regulate autoimmunity by impairing T cell activation and proliferation, and promoting Treg responses in rodents and humans^5^,^6^,^23^. MSCs can also secrete inhibitory cytokines, such as IL-10 and TGF-β1, which are potent inhibitors of T cell autoimmunity. In this study, we found that infusion with MSCs reduced the pancreatic insulitis and TNF-α expression, but increased the levels of IL-10 and TGF-β1 in the islets of diabetic NOD mice. Furthermore, infusion with MSCs increased the percentages of splenic Tregs and the levels of plasma IL-4, IL-10 and TGF-β1, but decreased the percentage of splenic CD8^+^ T cells and the levels of plasma IFN-γ, TNF-α and IL-17A in diabetic NOD mice. These data indicated that infused MSCs inhibited pathogenic Th1 and Th17 responses and promoted Treg responses in diabetic NOD mice, similar to that of previous observations^4^,^6^,^23^. More importantly, the modulatory effect of infused MSCs on T cell responses tended to be dose- and frequency-dependent. The increased levels of IL-10 and TGF-β1 in the islets may reflect the infiltration of Tregs, which inhibited pathogenic Th1 and Th17 responses to mitigate their destruction of islet β-cells and to preserve the β-cell function in diabetic NOD mice.

Moreover, the increased islet β-cell function may stem from MSC-promoting progenitor cell differentiation into insulin producing cells^12^,^19^. Alternatively, the infused MSCs may differentiate into insulin-producing cells or create a micro-environment favorable for residual β cell survival and function in diabetic NOD mice^24^,^25^,^26^. We are interested in further investigating the potential mechanisms underlying the action of infused MSCs in regulating T cell autoimmunity and preserving the β-cell function.

Previous studies have shown that infused MSCs can migrate to the inflammatory organs, including the islets^14^,^27^. In this study, we determined the dynamic distribution of infused MSCs and found that infused MSCs effectively accumulated in the pancreatic tissues at 4 weeks post infusion and were dramatically reduced at 6 weeks post infusion in NOD mice with severe T1D. In comparison with that in the mice receiving a single low dose of MSCs, infusion with a high dose of MSCs or multiple-dose of MSCs resulted in significantly greater numbers of infused MSC accumulation in the pancreatic tissues at 4 weeks post infusion in diabetic NOD mice. Interestingly, the levels of human Alu element DNA in the pancreatic tissues from the mice receiving multiple-dose of MSCs were significantly higher than that in those receiving a high-dose of MSCs at 6 weeks post infusion. Hence, the effect of MSC infusion on the accumulation of infused MSCs in the pancreatic tissues tended to be dose- and frequency-dependent and infusion with multiple-dose of MSCs caused longer presence of infused MSCs in the pancreatic tissues of severe diabetic NOD mice. These findings were consistent with a previous observation that infusion with multiple-dose of MSCs restores long-term glucose homeostasis in STZ-induced diabetic mice^15^. Therefore, infusion with multiple-dose of MSCs may be better to inhibit T cell autoimmunity and preserve β-cell function for treatment of severe T1D.

Our data indicated that infusion with bone marrow MSCs preserved β-cell function in some T1D patients with ketoacidosis and severe diabetic NOD mice. Infusion with MSCs promoted Treg responses and elevated the levels of islet and plasma IL-10 and TGF-β1, but decreased the levels of IFN-γ, TNF-α and IL-17A in diabetic NOD mice. The infused MSCs effectively migrated and accumulated in the pancreatic tissues of diabetic NOD mice. The therapeutic effects of MSC infusion tended to be dose- and frequency-dependent, and infusion with multiple-dose of MSCs held longer effects. Hence, infusion with MSCs, particularly with multiple-dose of MSCs may be valuable for intervention of T1D, even with diabetic ketoacidosis. We recognized that our studies had limitations, including very small sample size and the lack of functional studies of infused MSCs as well as the precise mechanisms underlying the action of infused MSCs in regulating T cell autoimmunity and preserving the β-cell function. Therefore, further studies are warranted to validate the findings and address the limitations in a bigger population.

## Methods

### Patients

A total of five T1D patients with ketoacidosis were recruited at the Division of Endocrinology, the Affiliated Drum Tower Hospital of Nanjing University Medical School (Nanjing, China) from March 2009 to May 2009. Individual T1D patients with ketoacidosis were diagnosed, according to the criteria of America Diabetes Association (ADA). All of the patients developed at least one of the glutamic acid decarboxylase antibody (GADA), protein tyrosine phosphatase antibody (IA-2A), islet cell antibody (ICA), and insulin autoantibody (IAA). Individual T1D patients were excluded if she/he had another autoimmune disease or had been treated with immunosuppressants. Written informed consent was obtained from individual subjects or their parents, and the experimental protocols were approved by the Ethics Committee of the Drum Tower Hospital Affiliated to Nanjing University. The methods used in the present study were carried out in accordance with the Declaration of Helsinki.

### MSCs Preparation and Transplantation

BM-derived MSCs were isolated from BM aspirates of healthy relatives of five patients. The cells were cultured and purified, as reported previously^28^. Briefly, mononuclear cells were isolated from heparinized human bone marrow samples (5 ml from each donor) by density-gradient centrifugation and cultured in 10% fetal bovine serum (FBS) DMEM for 48 h. After removal of non-adherent cell, the remaining cells were exposed to fresh medium every three days until the cell confluency. Subsequently, the cells were passaged. The cells at passage 2–5 were stained with fluorescent antibodies anti-human peridinin chlorophyll protein (PerCP)-CD45, phycoerythrin (PE)-CD14, fluorescein isothiocyanate (FITC)-CD29, FITC-CD44, PE-CD34, and PE-CD105 (eBioscience, San Diego, USA) and analyzed by flow cytometry using the BD FACSCanto™ flow cytometry system (BD BioSciences, San Jose, USA). A preparation of CD29^+^CD44^+^CD105^+^CD14^−^CD34^−^CD45^−^ MSCs with a purity of >95 was used for treatment.

### Treatment, Follow-up and Laboratory Tests

Individual patients were treated intravenously with MSCs at 1 × 10^6^ cells/kg body weight, besides regular insulin and anti-ketoacidosis treatments. All patients with MSC treatment complied with self-monitoring blood glucose and insulin injection and recorded insulin doses. They were followed up to four years post treatment and subjected to standard-meal glucose tolerance tests for the measurements of food-stimulated C-peptide. Their blood samples were obtained for the measurement of plasma HbA1c and C-peptide before and at 3, 6, 12 months and then yearly post MSC treatment. Individual patients, who achieved a decreased average daily insulin dosages more than 40% and fasting or postprandial C-peptide levels of less than 30% within six months were considered as responders.

### Mice and the Diabetic Model

Female NOD/Ltj and C57BL/6 mice at 7–8 weeks of age were purchased from the Animal Model Research Center of Nanjing University (Nanjing, China) and housed in a specific pathogen-free facility at a cycle of 12:12 light/dark, constant temperature and humidity. The experimental protocols were approved by the Nanjing University Medical School and were performed following the Guidelines of Institutional Animal Care and Use Committee of Nanjing University.

### Preparation of MSCs and NOD Mice Treatment

Written informed consent was obtained from healthy human subjects. MSCs were isolated, cultured and purified, as described previously. NOD/Ltj mice were injected intraperitoneally with 40 mg/kg *streptozotocin* (STZ, Sigma) for five consecutive days to accelerate diabetes development. Individual mice with blood glucose levels ≥20 mmol/L for 2 consecutive days were considered as severe diabetic. The diabetic mice were randomized and treated intravenously with vehicle Hank’s solution (200 μl) alone as the T1D control, 0.5 × 10^6^ MSCs in vehicle as the low dose, 1.0 × 10^6^ MSCs as the high dose or low doses of MSCs twice at an interval of one week as the multiple-dose groups. A group of C57BL/6 mice receiving corresponding buffers served as the healthy controls. Following treatment, their body weights were measured weekly and the levels of FBG and plasma insulin were examined longitudinally using the One Touch profile glucometer (Johnson & Johnson, New Brunswick, USA) or by ELISA using the Rat/Mouse Insulin ELISA kit, according to the manufacturers’ instruction (Millipore, Massachusetts, USA), respectively. At two, four and six weeks post treatment, some mice from each group were sacrificed and their tissues were dissected for the subsequent experiments.

### Plasma Cytokine Levels

The concentrations of plasma IFN-γ, IL-4, IL-10, TNF-α, IL-17A, and TGF-β1 in individual mice were measured by immunology multiplex assay in a MAGPIX system using the MT17MAG47K-PX25 and TGFBMAG-64K-03 kits (Millipore, Massachusetts, USA), according to the manufacturers’ instruction.

### Histology and Immunohistochemistry

At two, four and six weeks post treatment, some mice from each group were sacrificed and their livers, lungs, kidneys, small intestine and pancreatic tissues were harvested and weighed. The half of the pancreatic tissues was fixed and paraffin-embedded. The pancreatic tissue sections (3 μm) were stained with hematoxylin and eosin, and imaged under a Zeiss Axioplan light microscope. The contents of inflammatory infiltrates were scored in a blinded manner as 0 = no infiltration; 1 = infiltrates covering 1–10% of the islet area; 2 = 10–25% of the islet area; 3 = 25–50% of the islet area; and 4 = >50% of the islet area. At least 20 islets from each mouse pancreatic tissue were evaluated.

The contents of insulin in the islet β-cells were determined by immunohistochemistry. Briefly, the pancreatic tissue sections (5 μm) were deparaffinized, rehydrated and treated with 3% H_2_O_2_ in methanol for 15 min to inactivate endogenous peroxidase activity. The sections were subjected to antigen retrieval and treated with 2% bovine serum albumin (BSA) for 1 h. Subsequently, the sections were incubated with rabbit anti-insulin antibodies or control rabbit IgG (1:600; Santa Cruz Biotech, USA), and after being washed, the bound antibodies were detected with horseradish peroxidase (*HRP*)-conjugated goat anti-rabbit IgG (ZSGB-bio, Beijing, China), followed by visualizing with 0.2% 3,3′-diaminobenzidine (DAB). The integrated optical density (IOD) of anti-insulin staining in individual pancreatic tissues was evaluated using the Image-Pro Plus 6.0, (Planetron, Tokyo) and at least 20 islets selected randomly from each pancreatic tissue were analyzed.

The levels of TNF-α, TGF-β1, and IL-10 expression in individual pancreatic tissues were measured by immunohistochemistry. Briefly, the pancreatic tissue sections (5 μm) were incubated with rabbit anti-TNF-α, anti-TGF-β1, anti-IL10 or control rabbit IgG (1:200; Santa Cruz Biotech, USA) overnight at 4 °C, and the bound antibodies were detected with HRP-conjugated anti-rabbit IgG and visualized with DAB. After being imaged, the levels of anti-TNF-α, anti-TGF-β1 and anti-IL-10 staining in individual images were measured, and expressed as the ratios of total positively staining IOD to total areas imaged.

### Flow Cytometric Analysis

Splenic mononuclear cells were isolated and the frequency of CD4^+^, CD8^+^ and CD4^+^CD25^+^Foxp3^+^ Tregs in individual mice was determined by flow cytometry. Briefly, splenic mononuclear cells (1 × 10^6^/tube) were stained in duplicate with allophycocyanin (APC)-conjugated anti-CD3, FITC-anti-CD4, PE-anti-CD8 (eBioscience, San Diego, USA) and the percentages of CD4^+^ and CD8^+^ T cells were determined. Furthermore, splenic mononuclear cells were stained in duplicate with FITC-anti-CD4 and APC-anti-CD25, fixed, permeabilized and stained intracellularly with PE-anti-FoxP3 (eBioscience). The frequency of CD4^+^CD25^+^Foxp3^+^ Tregs in total CD4^+^ T cells was determined by flow cytometry. The data were analyzed with the FlowJo software.

### Preparation of Tissue Samples for Measuring the Alu Elements in Human DNA by Quantitative RT-PCR

To determine the dynamic distribution of infused MSCs, the dissected pancreatic tissues, livers, kidneys, lungs, and intestines were weighed, snap frozen in liquid nitrogen, and stored at −80 °C until use. The frozen liver, lung, kidney, small intestine and pancreatic tissues were homogenized, and their DNA was extracted using the Blood/Cell/Tissue Genome DNA Extract kit (TIANGEN, Beijing, China), according to the manufacturers’ instruction. After quantification of DNA concentrations, the levels of Alu DNA in individual samples were determined in triplicate by quantitative RT-PCR assay in StepOnePlus RT-PCR system (Applied Biosystems, Foster City, USA) using 100 ng sample DNA as the template, the Alu-specific primers, Taqman probe (Takara, Japan). The sequences of primers were: forward, 5′-CATGGTGAAACCCCGTCTCTA-3′; reverse, 5′-GCCTCAGCCTCCCGAGTA-3′; probe, 5′-FAM-ATTAGCCGGGCGTGGTGGCG-TAMRA-3′. The levels of human Alu DNA were calculated, according to the standard curve established by mixing different amounts of human genomic DNA with mouse monocyte genomic DNA.

### Statistical Analysis

Data are expressed as the mean ± SEM or individual values where applicable. The difference among groups was analyzed by ANOVA and post hoc Fisher’s least significant difference test. The potential associations between variables were analyzed by linear correlation using the SPSS software Window 15. A two-tailed *P*-value of <0.05 was considered statistically significant.

## Additional Information

**How to cite this article**: Li, L. *et al*. Infusion with Human Bone Marrow-derived Mesenchymal Stem Cells Improves β-cell Function in Patients and Non-obese Mice with Severe Diabetes. *Sci. Rep.* **6**, 37894; doi: 10.1038/srep37894 (2016).

**Publisher's note:** Springer Nature remains neutral with regard to jurisdictional claims in published maps and institutional affiliations.

## Figures and Tables

**Table 1:** The demographic and clinical characteristics of patients before treatment.

Patient no./sex	1/M	2/F	3/F	4/M	5/F
Age-onset (yr)	14.0	15.5	19.6	19.0	31.0
HbA1c (%)	6.1	5.9	7.2	9.5	13.1
Duration (month)	3.5	2.0	4.3	9.6	11.0
BMI (kg/m^2^)	15.8	18.3	20.1	19.9	19.8
DKA	Yes	Yes	Yes	Yes	Yes
Positive Ab	GADA	GADA/ICA	GADA	IA-2A	GADA
Insulin doses (IU/Kg/d)	0.6	0.4	0.4	0.7	0.5
FCP (pmol/liter)	390.9	334.1	318.6	28.8	284.1
PCP (pmol/liter)	940.5	978.9	937.4	32.1	744.2
Follow-up (month)	81.0	79.0	79.0	78.0	78.0

BMI, body mass index; DKA, diabetic ketoacidosis; FCP, fasting C-peptide; PCP, post-prandial C-peptide.
